# Powers of Entry under the Public Health (London) Act

**Published:** 1907-05-25

**Authors:** 


### POWERS OF ENTRY UNDER THE PUBLIC HEALTH (LONDON) ACT.

MR. PLOWDEN, the Marylebone magistrate, is facetious. He must have his joke, and, indeed, no one will grudge his habit of enlivening the dreary proceedings of the police court by the sparkle of his wit. That "the law is a hass," as was affirmed by Mr. Bumble, is a statement apparently endorsed by the mirthful magistrate. At all events, it has been left to Mr. Plowden to discover that the power of entry under the Public Health (London) Act is distinct from the power of egress, and that no power of exit is granted under the Act. On account of this ludicrous failure of the law, the Stipendiary Magistrate was unable to grant a warrant to empower the officials of the Marylebone Borough Council to enter a house in their borough for the purpose of removing the dust which had been accumulating there for several months past. The occupying tenants severally objected to the dust being carried through their respective tenements, and the magistrate, unable to grant a warrant empowering egress as well as entry, endeavoured to console the defeated officials of the Council with an expression of opinion that the first to suffer in health from the insanitary conditions would be the objecting parties themselves. Truly magisterial law is a wonderful instrument of public health administration.

